# The Burden of Migraine in Adults with Atrial Septal Defect: A Nationwide Cohort Study

**DOI:** 10.1038/s41598-019-43895-z

**Published:** 2019-05-15

**Authors:** Camilla Nyboe, Ann Hyldahl Nymann, Anne-Sif Ovesen, Vibeke Elisabeth Hjortdal

**Affiliations:** Department of Cardiothoracic and Vascular Surgery, Aarhus University Hospital, Aarhus, Denmark

**Keywords:** Congenital heart defects, Epidemiology

## Abstract

We aimed to investigate migraine diagnoses in a hospital setting, use of prescription migraine medicine and levels of serotonin in patients with atrial septal defect. Using Danish national registries to identify all patients born before 1994 diagnosed with atrial septal defect between 1959 and 2013, thus including 2277 patients and a gender and age matched comparison cohort of 22756. Plasma serotonin was measured in 136 patients with a small, unclosed, atrial septal defects and 18 controls. Patients with atrial septal defect had an increased risk of receiving a migraine diagnosis (HR 3.4 (95% CI: 2.6–4.6)) and receiving migraine medicine (HR 1.8 (95% CI: 1.2–2.5)). Ten years after closure, 93% of those using migraine medicine pre-closure, were still receiving this. The risk of having very high plasma serotonin levels was increased in patients with atrial septal defect compared with the control group, but there was no difference in the median values between the two groups. Migraine and use of migraine medicine were increased in atrial septal defect patients. The use of medicine was not diminished by closure of the defect. Plasma serotonin was severely elevated in 18% of the patients with atrial septal defect.

## Introduction

Migraine has been widely associated with right-to-left shunts and the incidence in patients with a persistent foramen ovale (PFO)^1^ is reported as high as 25–40%^2,3^. Several explanations have been suggested for this relation, without any clear identification of the cause. Platelet activation due to turbulent shear stress through the defect and thereby release of serotonin is believed to play a role in the pathogenesis^4^. Serotonin is mostly metabolised in the liver and lungs. With shunting through the atria, some of the serotonin rich blood is thought to bypass the liver and the lungs, resulting in theoretically higher levels of plasma serotonin. Serotonin causes both vasodilation and vasoconstriction in different concentrations, and can thereby be among the triggers of a migraine. Migraine has also been related to cerebral embolisms, and supports the theory of both platelet activation and shear stress induced by the embolism, as well as micro embolisms through the shunt as an independent activator of migraine, and platelet inhibitors have shown good results in symptom reduction in migraine patients with PFO^5–8^.

In patients with an atrial septal defect (ASD)^9^, the primary shunt direction is left-to-right. However, in some patients, there is a short time in each cardiac cycle where the shunt changes direction to right-to-left shunting, which can be prolonged in case of increased right side pressures i.e. during valsalva. Several studies have linked ASDs with migraine and found prevalences similar to that seen in right-to-left shunts^1,10–12^ with suggestions that size of the shunt influences the risk of migraine^13^. Whether closure of a shunt reduces the risk of migraine is uncertain. Some studies have found a reduction of the incidence of migraine^11,14^; some have found no change^12^ and yet some have found an increased risk of de novo migraine, but with no consensus on the continuance of this new onset migraine^1,12,15,16^. In this study we seek to investigate the burden of migraine in the ASD patients by investigating their risk of hospitalisation with a migraine diagnosis, the use of prescription migraine medicine both before and after closure and the levels of serotonin in patients with an unclosed shunt.

## Methods

### Study cohort and design

The Danish health care system is publicly funded and provides equal access to all Danes, who since 1968 have been provided with a unique personal identification number. Information on birth, emigration and date of death are all gathered in the Danish Civil Registration System^17^ and hospital data are transferred to several medical registries all linked by the identification number. This nation wide cohort study is based on information from three data sources. First, we included all patients registered with an ASD diagnosis in The Danish National Patient Registry (DNPR), which contains information on all in and out patient hospital contacts since 1977 including all diagnoses, dates of admission, dates of discharge and procedures performed. The diagnoses were coded according to the International Classification of Diseases (ICD) using the 8^th^ version until 1993 after which the 10^th^ edition was used. The ASD diagnosis of all patients identified in the registry was validated manually by review of medical records by two independent physicians. Patients were excluded if the defect was described as a Persistent Foramen Ovale; if the patient had a concomitant congenital heart disease; if the ASD diagnosis could not be confirmed in the hospital record or if the hospital record was lost and not available for validation. Only patients born before 1994 were included in this study. During this review, a small number of patients had an ASD diagnosis or closure performed before the initiation of the Danish national patient registry. These dates were corrected in the registry and thus, dates of diagnosis for included patients, ranges from 1959 and up to January 1 2013 even though the registry is only initiated in 1977.

Second, we included a separate population of ASD patients after validation of hospital records of patients under the age of 15 years given an ASD diagnosis in Danish hospitals between 1963 and 1974. Patients who did not survive to receive a personal identification number (n = 5) in this subgroup were excluded. The combined study population has previously been used in studies investigating mortality^18^, on going research on psychiatric disease and use of social benefits, and parts of the population has been used in investigating pneumonia^19^, and atrial fibrillation and stroke^20^.

Each ASD patient was matched with 10 subjects from the general Danish population on birth year and gender using the Danish Civil Registration System. The date of diagnosis of the ASD patient was used as a match date for the comparison cohort.

Finally, a subgroup from the ASD population, consisting of all patients with unrepaired ASD, were contacted for inclusion in a study on their general health. From this subpopulation, blood samples and questionnaire on subjective complaints of headache and migraine were collected from all included patients.

Information on closure or no closure was identified using procedure codes for surgical closure and transcatheter based closure in the DNPR. Both patients with closure, with no closure, and those who turned out to have spontaneously closed ASD on a control echocardiography, were included in the study.

### Clinical outcome

The DNPR was used to identify patients hospitalized or seen in out patient clinic due to the diagnosis migraine. All ICD-codes related to migraine were used as outcome (Appendix A). The DNPR, which contains information on prescription medicine for the entire Danish population, including ATC codes and date of dispensed prescription since 1994, was used to extract data on migraine prescription medication (Appendix B)^21^.

### Serotonin

Patients with no repair of their ASD (n = 136) and matched healthy controls (n = 18) had venous blood samples drawn by a trained nurse. All blood samples were handled with care, vials were not turned or shaken, and the blood samples were centrifuged within 20 minutes. Platelet Poor Plasma (PPP) was produced by centrifugation of whole blood by first 200 g in 10 minutes by 20 degrees Celsius after which the middle part of the plasma was removed and centrifugation was performed by 3000 g in 20 minutes and 4 degrees Celsius. The Plasma was stored at −80 degrees Celsius until analysis by High Pressure Liquid Chromatography was performed within 6 months. Serotonin was measured using tandem mass spectrometry. Cut off value for serotonin measurements were <10 nmol/L and reference value < 30nmol/L.

### Statistical analysis

Follow-up started at birth for both ASD patients and their control group. The date of the ASD diagnosis was used as an index date for matching the controls. The follow-up continued until time of event, death, emigration, or the end of follow-up (01-01-2018), whichever came first.

Cox proportional hazards regression was used to determine hazard ratios (HR) of receiving a migraine diagnosis for ASD patients, from birth or the beginning of the registry. The underlying time scale was age. Use of prescription migraine medication was analysed with the date of the ASD diagnosis as index date. The Danish Prescription Registry was not initiated until 1994 and therefore medicine prescribed before 1994 was unknown. Only patients diagnosed after 1994 were therefore included in the Cox regression analysis of prescription medicine. The HRs were adjusted for gender and stroke and we graphically verified the assumption of proportional hazards with log minus log plots. Direct comparison of the outcomes in the groups was analysed using an unpaired students t-test. Differences between plasma serotonin were not normally distributed and thus analysed using a Fisher’s one-tailed exact test.

All analyses were performed using Stata 15, StataCorp LP, Texas, USA.

### Ethical approval

All procedures performed in studies involving human participants were in accordance with the ethical standards of the institutional and/or national research committee and with the 1964 Helsinki declaration and its later amendments or comparable ethical standards. This study was approved by The Danish Data Protection Agency (j.nr. 2010-41-4649) and by the National Board of Health (j.nr. 7-604-04-2/193/KWH). Patients included for blood sampling all gave their informed written consent and this part of the study was approved by The Regional Committee on Biomedical Research Ethics of the Central Denmark Region (j.nr. M-2015-197-15). Due to restricted access to the Danish registries, no data are available for sharing.

## Results

We identified 4445 patients with an ASD diagnosed between 1977 and 2013 in the Danish National Patient Registry. We excluded 609 patients with concomitant congenital heart disease, and 651 where the hospital record was not available for validation. After validation of 3286 hospital records we excluded those with a PFO or no ASD found. Combined with the patients diagnosed between 1959 and 1977 (n = 253) the study population included 2277 patients with ASD along with 22,756 matched controls. The median follow-up time was 23.4 years (range 0.2–59.3 years). Most of the patients were female (60.8%) and the mean age at diagnosis was 26.7 years (±24.9) (Table 1).

**Table 1:** Baseline characteristics in patients with atrial septal defect and in the comparison cohort.

	ASD total	ASD closed	ASD no closure	Comparison cohort
**Baseline characteristics at time of ASD diagnosis**				
	(n = 2277)	(n = 1554)	(n = 723)	(n = 22756)
Female, n (%)	1384 (61%)	979 (63%)	405 (56%)	13834 (61%)
Hypertension, n (%)	262 (12%)	188 (12%)	74 (10%)	1661 (7%)
Diabetes, n (%)	116 (5%)	74 (5%)	42 (6%)	790 (4%)
Pulmonary heart disease, n (%)	123 (5%)	60 (4%)	63 (9%)	52 (0.2%)
Ischemic Heart Disease, n (%)	325 (14%)	239 (16%)	86 (12%)	1212 (5%)
Stroke, n (%)	180 (8%)	132 (9%)	48 (7%)	613 (3%)
Arrhythmia, n (%)	514 (22%)	396 (26%)	118 (16%)	715 (3%)

ASD: Atrial septal defect.

Of the 361 invited patients with no previous closure of the defect 136 ASD patients and 18 healthy controls were included for blood sampling. All patients had a trans thoracic echocardiography performed, which showed that 80% of the patients had a spontaneously closed ASD.

### Migraine

The ASD patients had an increased risk of receiving an in-hospital migraine diagnosis 54 (2.4%) of the ASD patients, and 154 (0.7%) of the comparison cohort), with a HR of 3.4 (95% CI: 2.5–4.6), (Table 2). This was the case for both patients without closure of the defect (HR 2.8; 95% CI: 1.8–4.2) and after closure of the defect in patients with no previous hospital admittance with migraine (HR 5.5, 95% CI: 3.4–8.9).

**Table 2:** Risk of receiving a migraine diagnosis in a hospital setting or receiving a prescription for migraine medicine in hazard ratios when compared to the comparison cohort.

	Risk of a migraine diagnosis *HR (95% Confidence Interval)*	Risk of using prescription migraine medicine *HR (95% Confidence Interval)*
ASD *total*	5.1 (3.6–8.47), p < 0.0001	1.8 (1.2–2.5), p = 0.0001
ASD *closed*	5.48 (3.37–8.89), p < 0.0001	1.6 (1.0–2.5), p = 0.06
ASD *not closed*	4.9 (2.13–11.27), p < 0.0001	2.3 (1.4–3.9), p < 0.0001

ASD: Atrial septal defect. HR: Hazard ratio.

### Medicine

In total 62 (2.74%) ASD patients received prescription migraine medicine, which was significantly higher than in the comparison cohort where 368 (1.62%) received medicine (p < 0.001). The overall risk of receiving prescription migraine medication was increased in the ASD patients compared with the comparison cohort with a HR adjusted for gender and stroke of 1.8 (95% CI: 1.2–2.5).

For patients with no closure, 22 received migraine medicine, which was significantly more than in the comparison cohort where 106, received migraine medicine (p = 0.001), HR 2.2 (95% CI: 1.4–3.5). In patients with closure of the defect, a total of 40 patients received migraine medicine. Of those, 14 did not receive medicine until after closure of the defect, whereas the rest all had migraine medicine prescribed before closure. The number was significantly higher than for the comparison cohort, where 262 received medicine (p = 0.011). The number of patients, who received migraine medicine, was almost unchanged 10 years after closure, where 37 still received migraine medicine (one had died). However, the risk of receiving migraine medicine after closure, for those with no previous history of migraine medicine prescriptions, was not statistically different from the comparison cohort (HR 1.44, 95% CI: 0.8–2.5).

### Serotonin

**Figure 1:**
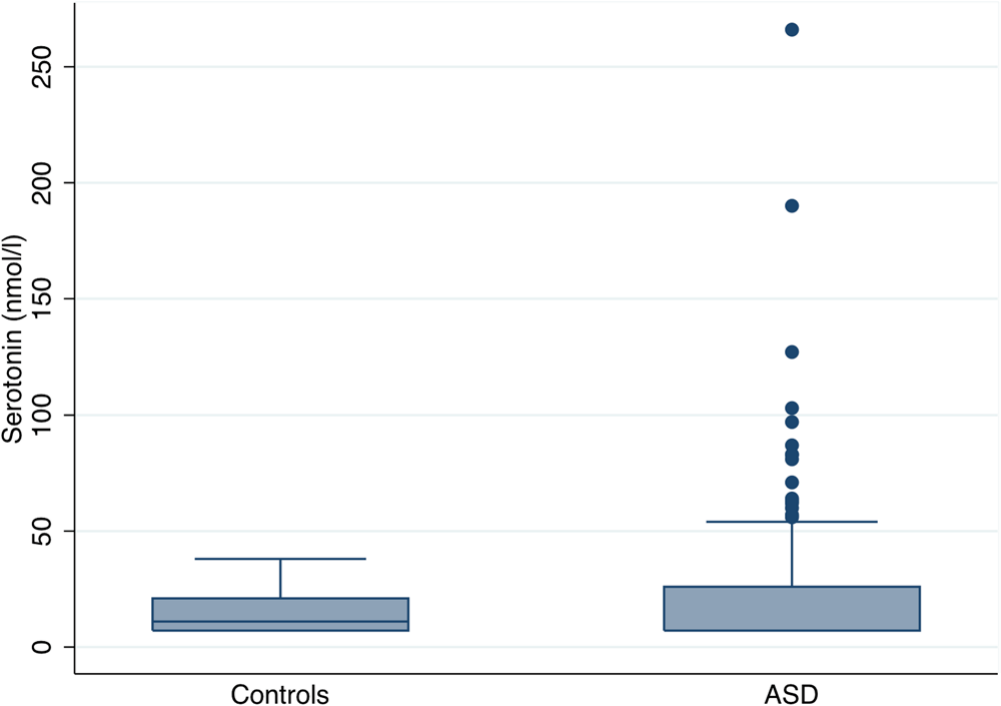
Levels of serotonin in atrial septal defect patients and the comparison cohort.

**Figure 2:**
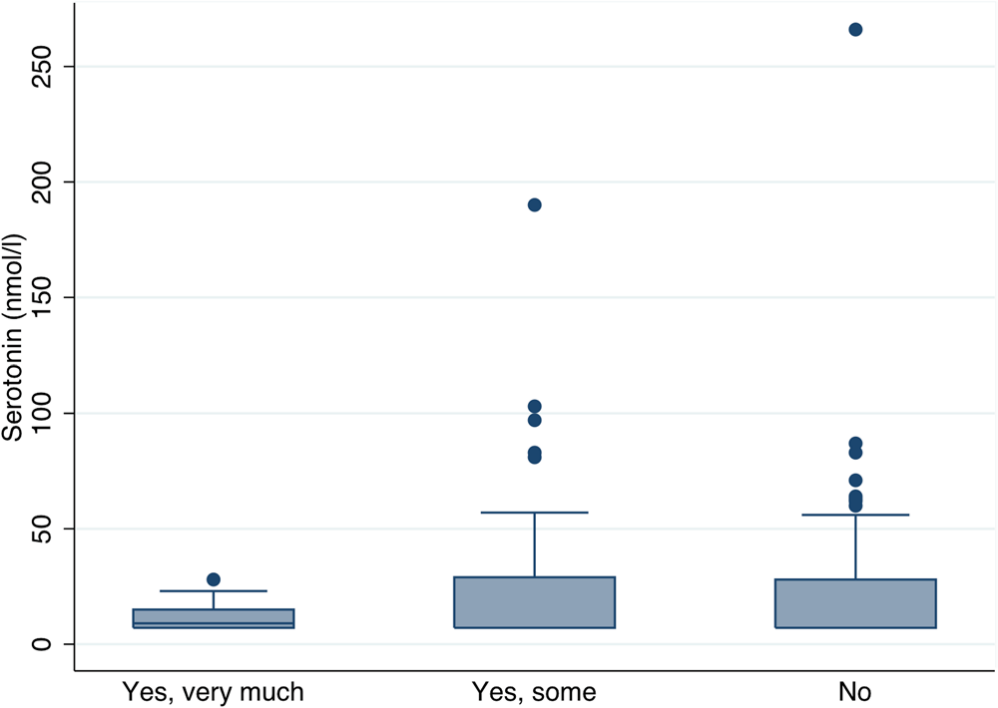
Serotonin levels in atrial septal defect patients with self reported symptoms of headache in groups of whether they are burdened with headache.

**Figure 3:**
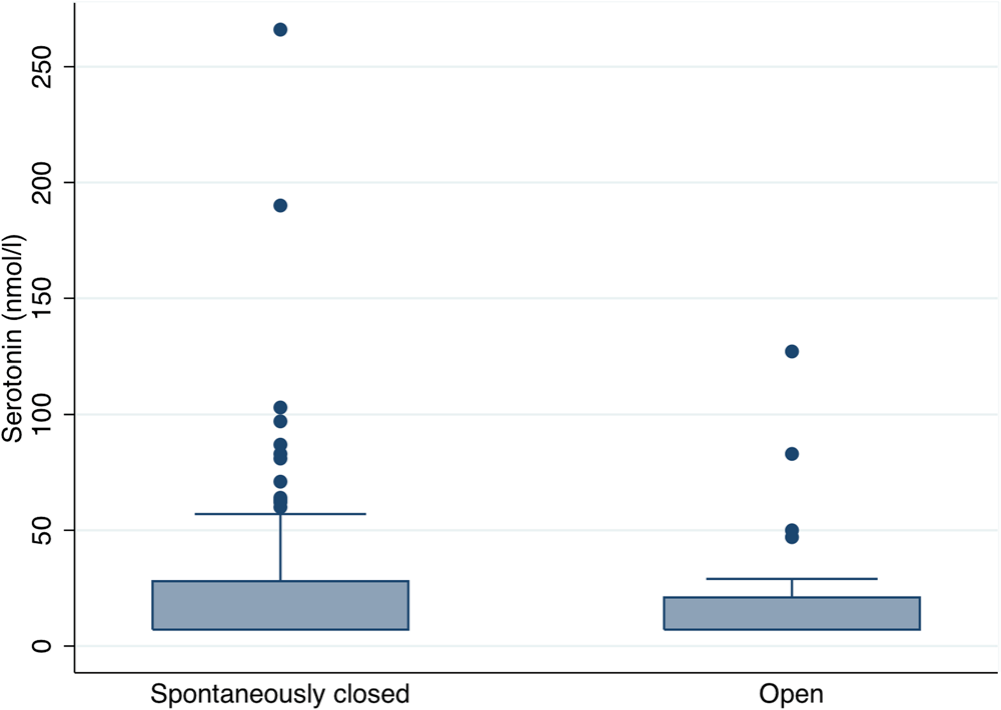
Levels of serotonin in atrial septal defect patients with open or spontaneously closed defects.

The serotonin levels were generally higher in the ASD patients, compared with the levels in the control group, although there was a no statistically significant difference between medians in the two groups; 11 nmol/L (7.09–38 nmol/L) in the controls versus 7.1 nmol/L (7.09–745 nmol/L) in the ASD group, p = 0.26). However, the risk of having very high values of serotonin was significantly higher in the ASD patients, with 24 patients (18%) having values above 40 nmol/L whereas none in the control group had values this high, p = 0.039 (Fig. 1). There was no correlation between having high serotonin levels and subjective complaints of headaches or migraine (Fig. 2), p = 0.12, and no significant difference in serotonin levels between those with spontaneously closed ASD and those with still open ASD (p = 0.21) (Fig. 3).

## Discussion

In this nationwide cohort study we found that patients with an ASD had an increased risk of receiving a migraine diagnosis compared with the comparison cohort, regardless of closure of the defect. The diagnoses are, however, all given in a hospital setting and are thus with a much lower prevalence than those reported in other studies^12,14^. The majority of migraine patients are self medicated or treated at their general practitioner, and they are rarely admitted to a hospital for their migraine. ASD patients could possibly be hospitalized more often with migraine as their primary diagnosis because of a high prevalence of migraine in the ASD population, or an increased severity of the migraine. A combination of these explanations could be true and a higher prevalence of migraine is in line with studies finding the risk significantly increased for both patients with a PFO or ASD^1,10,11^.

The increased risk of using prescription migraine medicine in the ASD patients supports the finding that these patients have either; a higher prevalence of migraine, or the severity of the migraine is increased, thus requiring medicine more often than the comparison cohort. Whether a closure of the defect relieves the patient of the migraine is uncertain. Luermans *et al*.^11^ found that the increased migraine prevalence in 68 ASD patients decreased after closure, which was also found by Azarbal *et al*. where 75% of patients with migraine were relieved of symptoms after closure^14^. Several studies have reported findings of new-onset migraine after closure with no cessation of the migraine at end of follow-up^1,15,22^ whereas Voet *et al*. found that new onset migraine was limited to the first years post closure^12^. This may indicate that some patients with migraine will benefit from getting their defects closed, but some patients might develop new-onset migraine after closure. In our study, the risk of receiving prescription migraine medicine was not significantly increased in patients with no history of migraine before closure when compared with the background population, indicating that the risk of severe de novo migraine might not be increased after closure. However, patients receiving migraine medicine is only the top of the iceberg and less severe migraine could be increased in these patients. Patients using migraine medicine prior to closure did not seem to achieve freedom from medication and almost all the patients, including those with new-onset medicine use after closure, still used prescription migraine medicine ten years after closure. This suggests that patients with severe migraine does not benefit from closure of their ASD in regard to severe migraine.

In general, the serotonin levels were not significantly increased in the ASD patients compared with the control group in this study. We did, however, find a number of ASD patients with very high levels of serotonin. The high levels of serotonin failed to show an association with subjective reporting of migraine or headaches in our patients, suggesting that even though ASD patients might have an increased risk of very high levels of serotonin, this is not related to their increased risk of severe migraine. The susceptibility for serotonin is influenced by gender, alcohol consumption, food and medication and was not adjusted for in this study, which could explain the lack of relation between serotonin and migraine that are seen in other studies^4,23,24^. The serotonin levels measured in this study are somewhat higher than seen in some studies but within normal reference values in other studies^25^. The patients included for blood sampling were all part of a study of patients with small and unclosed ASDs and 80% of the patients had a spontaneously closed ASD on transthoracic echocardiography. A trans oesophageal echocardiography could possibly detect smaller defects in these patients, but was not performed due to patient acceptance and ethics. The number of spontaneous closures is likely overestimated, and we do not know the magnitude of occasional right-to-left shunting in these patients. We did not find any difference in high levels of serotonin between those with spontaneously closure and those with an open defect. Size of the defect has been related to the severity of migraine^13^. The tested patients with open defects all had small to medium sized defects and measurements in patients with large ASDs prior to closure would possibly show even higher values and a relation to migraine.

### Limitations

Several limitations must be considered interpreting register-based data. There is a lack of knowledge on size, type and location of the ASD from the registries and this information are also sparse in the hospital records. However, due to validation of all hospital records it was possible to only include ASD patients and not patients with a PFO, which strengthens this study. The fact that only patients with severe migraine are admitted to hospital and receive a diagnosis for their migraine in a hospital setting limits this study to a small group of the most affected patients. The use of migraine medicine adds to the number of identified migraine patients but it is probably only the tip of the iceberg and we cannot provide the actual prevalence of migraine in the ASD population. Patients with an ASD are hospitalized at least once and often several times due to their congenital heart disease. The risk of being diagnosed with migraine when symptoms appear could be elevated compared with patients who might never be hospitalized, thus overestimating the risk of migraine in the ASD patients.

Patients with an ASD have an increased risk of receiving a hospital related migraine diagnosis, of receiving prescription migraine medicine and have a higher risk of having very high plasma serotonin levels when compared with a gender and age matched comparison cohort. The risk of using migraine medicine is not reduced 10 year after closure. Patients with no previous use of migraine medicine do not have increased risk of using prescription migraine medicine after closure compared with the comparison cohort. Migraine was not associated with very high levels of serotonin in the patients with a small or spontaneously closed ASD.

## Supplementary information

Appendix
