# Supplementary material for: The Burden of Migraine in Adults with Atrial Septal Defect: A Nationwide Cohort Study
Source: Sci Rep. 2019 May 15;9:7410. doi: 10.1038/s41598-019-43895-z (PMC6520354; doi:10.1038/s41598-019-43895-z)
Supplement: Supplementary file 1 — Appendix [file 41598_2019_43895_MOESM1_ESM.pdf]

The Burden of Migraine in Adults with Atrial Septal Defect: A Nationwide Cohort Study

Camilla Nyboe MD PhD; Ann Hyldahl Nymann MD; Anne-Sif Ovesen MD; Vibeke Elisabeth Hjortdal MD PhD

Appendix A

Diagnosis	ICD-8	ICD-10
Migraine	34600 Hemicrania ophthalmoplegica<br>34601 Hemicrania neuralgiformis periodica (Horton)<br>34608 Hemicrania alia definita<br>34609 Hemicrania	DG43 – Migraine<br>DG430 – Migraine without aura<br>DG431 – Migraine with aura<br>DG431A – Aura without headache<br>DG431B – Migraine equivalents<br>DG431C – Hemicrania hemiplegica famillaris<br>DG432 - Status migrainosus<br>DG433 – Migraine with complication<br>DG433A - Migraine with cerebral infarction<br>DG433B - Migraine with hemiplegi<br>DG438 – Other form of migraine<br>DG438A – Hemicrania ophthalmoplegica<br>DG438B – Hemicrania reinalis<br>DG438C - Atypical migraine<br>DG439 - Migraine UNS<br>DZ033B - Observation because of possible migraine

Appendix B

Diagnosis	ICD-10
Migraine medication	N02C - Medication against migraine<br><br>N02CA - Ergot-alkaloids<br><br>N02CA01 - Dihydroergotamin<br><br>N02CA02 - Ergotamin<br><br>N02CA04 - Methysergid<br><br>N02CA06 - Dihydroergocristin<br><br>N02CA07 - Lisurid<br><br>N02CA51 - Dihydroergotamin, combi<br><br>N02CA52 – Ergotamin, combi<br><br>N02CA56 – Dihydroergocristin, combi<br><br>N02CA72 - Ergotamin and with psycholeptica<br><br>N02CB – Corticosteroid-derivatives<br><br>N02CB01 - Flumedroxon<br><br>N02CC - Selective 5ht(1)-receptor-agonists<br><br>N02CC01 - Sumatriptan<br><br>N02CC02 - Naratriptan<br><br>N02CC03 - Zolmitriptan<br><br>N02CC04 - Rizatriptan<br><br>N02CC05 - Almotriptan<br><br>N02CC06 - Eletriptan<br><br>N02CC07 - Frovatriptan<br><br>N02CX - Other migraine medication<br><br>N02CX01 - Pizotifen<br><br>N02CX02 - Clonidin<br><br>N02CX03 - Iprazochrom<br><br>N02CX05 - Dimetotiazin<br><br>N02CX06 – Oxetoron
